# Elucidating the Role of Bacterial and Arbuscular Mycorrhizal Fungi Inoculants in Mitigating Nitrous Oxide (N_2_O) Emissions in Agroecosystems Under Climate Change

**DOI:** 10.3390/microorganisms14071412

**Published:** 2026-06-27

**Authors:** Ahmed M. El-Sawah, Ghada G. Abdel-Fattah

**Affiliations:** 1 Department of Agricultural Microbiology, Faculty of Agriculture, Mansoura University, Mansoura 35516, Egypt; 2 Department of Botany, Faculty of Science, Mansoura University, Mansoura 35516, Egypt; ghabdelfattah@mans.edu.eg

**Keywords:** denitrification, N_2_O mitigation, *nosZ* gene, nitrification, nitrogen cycle, soil salinity

## Abstract

Nitrous oxide (N_2_O) is a greenhouse gas that has a global warming potential approximately 300 times that of carbon dioxide (CO_2_). It is largely produced in agricultural soils through nitrification and denitrification processes driven by specific microbial functional genes (e.g., *amoA*, *nirS*, and *nirK*), which represent the main source of its emissions. The intensive use of nitrogen fertilizers increases nitrogen surplus in the ecosystem. This in turn accelerates the risk of nitrogen loss through leaching and volatilization, while also accelerating microbial pathways that drive N_2_O emissions in the soil. This issue raises severe environmental concerns within the context of global climate change, particularly through the climate-driven escalation of soil salinity, which further alters the microbial community and increases these emissions. Microbial inoculants, including bacteria and arbuscular mycorrhizal fungi, provide eco-friendly biological solutions to mitigate N_2_O emissions from agricultural soils. These inoculants could restore nitrogen balance in the soil by several strategies, such as improving nitrogen use efficiency, competing with native nitrifiers, and upregulating *nosZ* gene expression. This review highlights the current developments in the utilization of microbial inoculants for N_2_O mitigation, focusing on key bacterial genera (e.g., *Bradyrhizobium*, *Dyadobacter*, *Stutzerimonas*, *Paenibacillus*, and *Bacillus*) and arbuscular mycorrhizal fungi (AMF, e.g., *Rhizophagus* and *Funneliformis*), as well as the mechanisms used by these microorganisms. It also discusses the potential of using microbial inoculants in saline-affected soils, as well as the link between salinity and N_2_O emissions. Based on these insights, this review presents a thorough framework for the prospective use of microbial inoculants as an effective solution to sustainable agriculture while reducing the environmental hazards associated with N_2_O emissions, which endanger global food and climate systems.

## 1. Introduction

Nitrous oxide (N_2_O) is a greenhouse gas produced during the nitrogen cycle in agricultural soils [1]. Although N_2_O is less common than carbon dioxide (CO_2_), its capacity to cause global warming is 300 times greater [2]. Currently, the global atmospheric N_2_O levels have crossed 339.75 parts per billion (ppb), with annual increases ranging from 1.02 to 1.24 over the last two years [3]. This increase is due to anthropogenic activities, which are predicted to accelerate global warming while remaining the major cause of ozone depletion. Considering the atmospheric levels of N_2_O, approximately 70% of its overall emissions are driven by agricultural soils [4]. The production of N_2_O in the agricultural soil is a biological process, carried out by microorganisms within the nitrogen cycle through the nitrification and denitrification processes, and this is affected by specific factors, such as nitrogen availability and other environmental factors [5,6,7].

Microbial inoculants, such as bacteria and arbuscular mycorrhizal fungi (AMF), have the potential to mitigate N_2_O emissions in agroecosystems through a variety of strategies. For instance, bacterial inoculation can drive this mitigation through different pathways [8]. (1) Inoculation with *nosZ* gene bacteria allows for the direct reduction of N_2_O into N_2_ while also reducing the denitrification activity of indigenous microorganisms by outcompeting indigenous denitrifying microorganisms for electron donors (organic compounds) and acceptors (NO_3_^−^ and NO_2_^−^). The *nos*Z gene encodes the enzyme nitrous oxide reductase (N_2_OR), which converts N_2_O to harmless nitrogen gas (N_2_). (2) Inoculation with non-N_2_O-reducing microorganisms that modify the native microbial community’s structure and activity, which are involved in N_2_O production and/or reduction. Along with these bacterial actions, AMF play a key role in reducing N_2_O emissions through several mechanisms, such as improving nitrogen use efficiency and outcompeting nitrifiers for ammonium substrates [9]. This competition is driven by the fact that AMF are capable of transferring substantial quantities of nitrogen to host plants via their hyphal network [10]. In this line, Storer et al. [9] reported that AMF hyphae sequester ammonia from soil, and this reduced nitrification-driven N_2_O by outcompeting nitrifiers for substrate. However, the efficiency of AMF in the mitigation of N_2_O is strongly modulated by environmental stress and climatic variability [11]. For instance, Li et al. [11] found that the capacity of AMF to mitigate N_2_O emissions significantly decreases under severe drought and precipitation variability. Therefore, selecting the appropriate AMF strains and thoroughly assessing site-specific characteristics and environmental fluctuations are very important to optimize mitigation outcomes and ensure consistent field performance.

Soil salinity has been significantly exacerbated by global climate change, posing a global threat to agriculture. Soil salinity, at a certain level, inhibits the nitrification process, leading to nitrogen loss through ammonia volatilization and nitrous oxide emissions [12]. A study by Reddy and Crohn [13] revealed that active organic amendments can reduce N_2_O emissions in salt-affected soils by promoting complete denitrification to N_2_ via enhanced microbial activity, albeit this effect is temporary and may diminish over time as the organic matter stabilizes. Given these challenges, introducing specialized microbial inoculants can aid in mitigating N_2_O in saline-affected soils. A recent study by Li et al. [14] found that microbial inoculants such as *Bacillus subtilis* could reduce N_2_O emissions in saline-alkali soils by suppressing nitrification-related N_2_O production pathways.

This review focuses on the studies that documented the role of microbial inoculators, including both bacteria and arbuscular mycorrhizal fungi, in mitigating N_2_O emissions in agroecosystems and discusses the latest research on their underlying mechanisms. However, current literature is largely biased toward bacterial inoculants, leaving huge knowledge gaps about the role of fungal inoculants, such as arbuscular mycorrhizal fungi (AMF). To address this gap, this review expands the scope to include AMF. It also shifts the focus toward salt-affected soils by highlighting the link between salinity and N_2_O emissions and the potential use of microbial inoculants in saline-affected soils for reducing N_2_O emissions. Specifically, this review addresses these questions: (1) What is the role of arbuscular mycorrhizal fungi (AMF) in mitigating N_2_O emissions? (2) How does soil salinity, driven by climatic change, affect N_2_O emission dynamics? (3) Can microbial inoculants effectively reduce these emissions in salt-affected soils? Hence, this review provides a thorough framework for employing microbial inoculants as a sustainable agricultural solution for mitigating climate-threatening N_2_O emissions.

## 2. Methodology

To provide an objective overview of current developments, this review includes peer-reviewed literature published up to 2026. The search focused on electronic databases such as Web of Science, Scopus, and Google Scholar using keywords related to microbial inoculants, nitrous oxide mitigation, soil salinity, and N-cycling genes. Studies were chosen based on their relevance to mechanistic pathways used by bacteria and arbuscular mycorrhizal fungi in the mitigation of N_2_O emissions to create a clear framework for evaluating how bioinoculants decrease agricultural N_2_O.

## 3. Nitrification in Agricultural Soils

Nitrification is an important process in agricultural soils and strongly affects nitrogen transformations and nitrogen use efficiency. It is a process carried out by chemolithotrophic bacteria through the oxidation of nitrogen molecules in two steps [8]. In the first step, ammonia (NH_4_) is oxidized to nitrite (NO_2_) using the ammonia monooxygenase enzyme (AMO) by ammonia-oxidizing bacteria (AOB) such as *Nitrosomonas*, *Nitrosococcus*, *Nitrosospira*, *Nitrosovibrio*, *Nitrosolobus,* and ammonia-oxidizing archaea (AOA). In the second step, nitrite (NO_2_) is oxidized to nitrate (NO_3_) by nitrite-oxidizing bacteria (NOB) such as *Nitrobacter*, *Nitrococcus*, and *Nitrospira*. Nitrification then provides nitrate, the most oxidized form of nitrogen required for plant uptake. However, the resulting nitrate is also susceptible to other processes such as nitrogen loss through leaching and denitrification.

In addition, the recent discovery of complete ammonia oxidation (comammox) has updated our concept of nitrification as a two-step process [15,16]. Comammox bacteria, which belong to the genus *Nitrospira,* can oxidize ammonia directly to nitrate within a single step [16]. This discovery changes our understanding of the nitrogen cycle in nature [17].

## 4. Denitrification in Agricultural Soils

Denitrification is a crucial process within the nitrogen cycle, in which nitrates (NO_3_) are gradually reduced to atmospheric nitrogen gas (N_2_) under anaerobic conditions by heterotrophic, facultative anaerobic bacteria [8]. This biological process involves four stages [18]: (1) Nitrate (NO_3_^−^) to Nitrite (NO_2_^−^): The first stage involves the reduction of NO_3_ to NO_2_ by nitrate reductase enzymes, which can be either membrane-bound (Nar) or periplasmic (Nap). (2) Nitrite (NO_2_^−^) to Nitric Oxide (NO): The second stage involves the reduction of NO_2_ into gaseous nitric oxide (NO) by nitrite reductase enzymes typically encoded by the *nirS* or *nirK* genes. (3) Nitric Oxide (NO) to Nitrous Oxide (N_2_O): The third stage involves the transformation of NO into nitrous oxide (N_2_O) by nitric oxide reductase (Nor). (4) Nitrous Oxide (N_2_O) to Nitrogen gas (N_2_): The fourth stage involves the reduction of N_2_O into atmospheric nitrogen (N_2_) by the enzyme nitrous oxide reductase (*nosZ*), encoded by the *nosZ* gene. The overall progression of these redox reactions can be represented as: (NO_3_^−^ → NO_2_^−^ → NO → N_2_O → N_2_). However, under field conditions, this sequential pathway is highly susceptible to incomplete denitrification and N_2_O accumulation, driven by environmental factors such as fluctuating oxygen dynamics, soil pH, and C/N ratio acting through the microbial community [19]. In addition to denitrification, the anaerobic ammonium oxidation (anammox) pathway serves as another route for N_2_ production by coupling the oxidation of ammonium with the reduction of nitrite to form N_2_ by passing the production of the N_2_O intermediate [20]. Regarding the final step of denitrification, there are two major clades in *nosZ* phylogeny: clade I and clade II [21,22,23]. Both clades regulate N_2_O gas emissions. Clade I is typically found in complete denitrifiers, which manage nitrogen turnover by both producing and consuming N_2_O. Conversely, Clade II is predominantly carried by non-denitrifying bacteria that act strictly as N_2_O sink without contributing to its production.

## 5. Nitrous Oxide (N_2_O)

**Figure 1:**
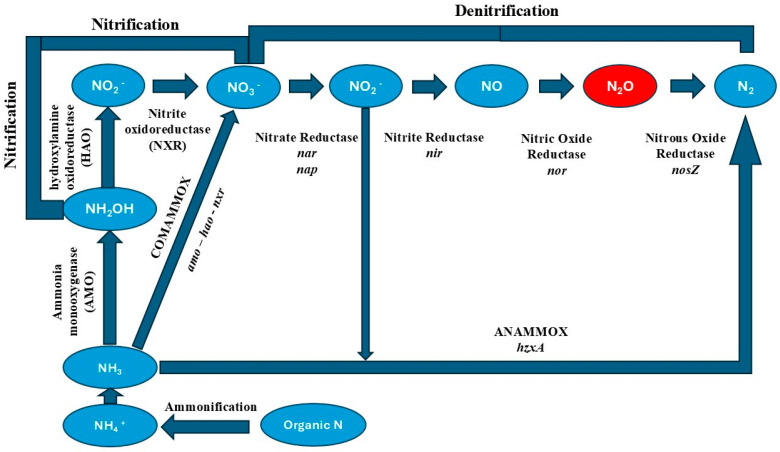
Conceptual diagram of microbial nitrogen transformation pathways and the key functional genes involved in N_2_O production.

Nitrous oxide (N_2_O) is a major greenhouse gas that has the potential to cause global warming more than carbon dioxide (CO_2_) by about 300 times [24]. Excessive use of nitrogen fertilizers is the primary cause of N_2_O emission from agricultural soil, accounting for approximately 60% of global anthropogenic N_2_O emissions [25]. Soil N_2_O is primarily produced through the processes of nitrification and denitrification [26]. During nitrification, the ammonia-oxidizing bacteria and archaea yield N_2_O as a byproduct through the oxidation of hydroxylamine to NO_2_^−^ [27]. In denitrification, N_2_O is released as an intermediate during the reduction of nitrate to N_2_ [28]. N_2_O emissions vary highly depending on soil type, environmental conditions, and management practices [29,30]. The *nirS* and *nirK* genes encode nitrite reductases that are involved in the synthesis of N_2_O, while the *nosZ* genes encode N_2_O reductases that are involved in the reduction of N_2_O to N_2_ [31]. Therefore, the ratio of (*nirS* + *nirK*)/*nosZ* has been used as an indicator for N_2_O emission potential [32]. The conceptual relationship between the microbial transformation pathways and the key functional genes involved in N_2_O production is summarized in Figure 1.

## 6. Bacterial-Inoculant-Mediated N_2_O Mitigation

The excessive use of chemical nitrogen fertilizers combined with low nitrogen use efficiency in the agricultural ecosystem resulted in considerable N_2_O emissions from soils [33]. Hence, the integration of microbial inoculants in agricultural practices is no longer optional but necessary to bridge the gap between high productivity and environmental preservation. Table 1 presents a summary of several bacterial inoculants, mitigation strategies, and underlying mechanisms of N_2_O mitigation. The following sections describe the direct and indirect strategies of bacterial inoculants to mitigate N_2_O emissions:

### 6.1. Direct Strategy

Using bacteria that produce the *nosZ* enzyme directly can provide a biological and effective solution for reducing nitrous oxide (N_2_O) emissions from agricultural soils, as this enzyme converts nitrous oxide (N_2_O) into nitrogen gas (N_2_) [8]. Indeed, field studies have confirmed the effectiveness of this concept, showing that inoculation of *nosZ*+ and non-genetically modified organism *nosZ*++ strains of *Bradyrhizobium japonicum* significantly mitigates N_2_O emissions released from decomposing root nodules of the soybean ecosystem at the post-harvest phase [34]. While previous studies relied on the use of highly active mutants (*nosZ*++), recent studies have focused on using indigenous strains (*nosZ*+) as a more sustainable alternative to overcome obstacles regarding the use or acceptance of mutated or genetically modified strains. At the field scale, Akiyama et al. [35] showed that N_2_O emission was reduced by inoculation with a mixed culture of indigenous *nosZ*+ strains of *Bradyrhizobium diazoefficiens* USDA110 group isolated from Japanese soils. This could be a more feasible and cost-effective biological solution for global soybean-producing countries than generating mutants. Another study by Hénault et al. [36] found a remarkable reduction in N_2_O emissions (70%) using the strain (*nosZ*+ G49) when compared to the (*nosZ*− USDA138) strain in association with soybean. Beyond symbiotic rhizobia, a recent study highlights the potential of the non-denitrifying bacterium *Dyadobacter fermentans* NS114^T^ to act as N_2_O sinks. This bacterium could enhance the soil’s capacity to consume N_2_O produced by other microorganisms in the soil by utilizing N_2_O reductase NosZII. N_2_O emissions were reduced by up to 189% in more than 1/3 of the soils (11 different soils), depending on soil pH and chemical properties [37].

The variability in N_2_O mitigation percentages among direct strategies presented in Table 1 (ranging from 70% to 189%) is directly governed by differences in experimental scales, soil properties, and microbial adaptation. Despite the success of the previous greenhouse and microcosm experiments, there are some limitations to their widespread use as reproducible agricultural tools. For example, while *Dyadobacter fermentans* exhibited high mitigation efficacy (up to 189%) across different soil types [37], its efficacy can be highly sensitive to moderate and high soil acidity, rendering inoculation ineffective in acidic soils. Moreover, the strategy based on rhizobia bacteria is limited to a specific plant and growth stage [34,35] and may be unable to control the increase in N_2_O emissions during fertilization in other growing seasons. Furthermore, the inoculated strains may fail to compete with existing soil microbial communities, resulting in reduced survival and effectiveness when transferred from controlled experimental plots to diverse soil types. Therefore, future studies should focus on building a tailored microbial inoculum consortium that can survive and maintain its viability and effectiveness in different agricultural soil systems.

### 6.2. Indirect Strategy

Inoculation with non-N_2_O-reducing microorganisms can indirectly mitigate N_2_O emissions by modulating the soil’s ecology. In this case, microbial inoculation contributes to suppressing N_2_O production or enhancing its reduction by altering the composition and the metabolic functions of indigenous microbial communities [8]. A study conducted by Gao et al. [38] indicated that inoculation with *Stutzerimonas stutzeri* (strains NRCB010 and NRCB025) improved tomato growth and reduced N_2_O emissions by up to 76.6%, particularly in coarse-textured soil. This mitigation is attributable to the change in soil microbial communities and the optimization of functional gene ratios, notably reducing the (*amoA* + *amoB*)/(*nosZI* + *nosZII*) and (*nirS* + *nirK*)/(*nosZI* + *nosZII*) ratios. These findings show that the efficacy of PGPR-based biofertilizers is influenced by soil texture, pH, and organic matter content, highlighting the necessity for site-specific biofertilizer applications. According to Zhou et al. [39], while urea application can increase N_2_O emissions in tea fields by up to 973.7 times over the control, the integration of *Paenibacillus polymyxa* biofertilizer mitigates this impact by 36.5–73.1% by stimulating the abundance of *nirK* and *nosZ* genes, which enhances natural denitrification and converts harmful greenhouse gases into harmless nitrogen. Similarly, *Bacillus amyloliquefaciens* has been shown to achieve a 50% reduction in N_2_O emissions in acidic vegetable soils by inhibiting ammonia-oxidizing bacteria and promoting the abundance of N_2_O-reducing bacteria, while simultaneously enhancing plant growth and soil pH [40].

**Figure 2:**
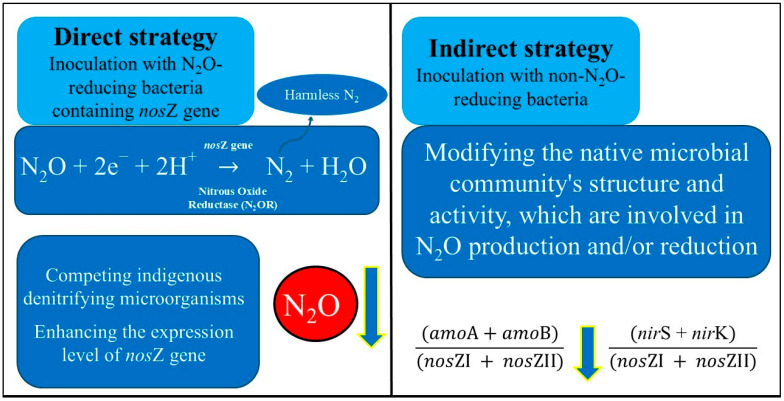
Conceptual diagram showing direct and indirect strategies of bacterial inoculants for mitigating N_2_O in agricultural soils.

Mitigation of N_2_O emission by indirect strategy in Table 1 displays a different range of efficacy, ranging from 36.5% to 76.6% (e.g., 50% for *Bacillus amyloliquefaciens* in acidic soils [40]). Despite the success of the previous microbial strains in mitigating N_2_O emissions, their transferability to open agroecosystems remains restricted by their dependence on controlled environments or in situ conditions. This underscores the critical need to evaluate soil properties before large-scale applications to produce a tailored inoculum and ensure its survival and vitality. A Conceptual diagram showing direct and indirect strategies of bacterial inoculants for mitigating N_2_O in agricultural soils is presented in Figure 2.

## 7. Arbuscular Mycorrhizal Fungi (AMF)-Inoculant-Mediated N_2_O Mitigation

Arbuscular mycorrhizal fungi (AMF) are critical beneficial soil microorganisms for sustainable agriculture, playing a vital role in mitigating nitrous oxide (N_2_O) within agroecosystems [9]. Using ^15^N_2_ isotope labeling [41], researchers can ensure that the nitrogen absorbed by the plant is the same as that applied as fertilizer, and that ammonium-based fertilization, when combined with AMF symbiosis, is more effective than nitrate-based inputs in enhancing plant biomass while minimizing gaseous N losses. AMF are suggested to mitigate N_2_O emissions through two synergistic pathways: (1) optimizing root morphology by increasing root length and surface area to enhance ^15^N_2_ uptake and deplete available soil N substrates and (2) altering the community of soil denitrifiers to favor a lower (*nirK* + *nirS*)/*nosZ* gene ratio by increasing the relative abundance of nirS-*Bradyrhizobium* and *Rubrivivax* with ammonia input, but reducing nosZ-*Azospirillum*, *Cupriavidus,* and *Rhodopseudomonas* under both fertilizer inputs. Furthermore, N_2_O emissions were significantly and positively correlated with the nosZ-type *Azospirillum*, *Cupriavidus,* and *Rhodopseudomonas* but negatively correlated with the nirS-type *Bradyrhizobium* and *Rubrivivax*. Table 2 presents a summary of several AMF inoculants, mitigation strategies, and underlying mechanisms of N_2_O mitigation. According to Wang et al. [31], AMF (*Rhizophagus intraradices* BJ09 and *Funneliformis mosseae* BJ04A), especially when combined with biochar, increase maize biomass by up to 19.23% while reducing soil inorganic nitrogen by 75.56%. This combination also suppresses N_2_O emissions, which correlates with a shift in the balance of denitrification functional genes (reducing the *nirS* + *nirK*/*nosZ* ratio) and increasing soil N unitization efficiency. Moreover, AMF potentially mitigate N_2_O emissions by competing with nitrifying microorganisms for available nitrogen. A study conducted by Storer et al. [9] found that AMF hyphae effectively sequester ammonium (NH_4_^+^), thereby reducing the substrate available for nitrification-driven N_2_O production. This competitive advantage is especially noticeable after ammonium application, as the presence of AMF sustains low N_2_O flux as compared to non-mycorrhizal soils. According to the study of Li et al. [11], AMF may act as a biological buffer against climate change in temperate grasslands. AMF were shown to be associated with reduced N_2_O emissions throughout a wide range of precipitation levels. Their relative contribution increases when water availability declines, except under extreme drought conditions (−70%). The microbial process underlying this reduction employs a two-pronged approach: (1) AMF considerably reduce the concentration of soil available nitrogen; (2) AMF are associated with shifts in the composition of the soil bacteria community, particularly those linked with N_2_O production. AMF hyphal density has a negative correlation with N_2_O-producing genes (*nirK* and *nirS*) and is positively correlated with N_2_O-consuming genes (*nosZ*).

In large-scale agroecosystems, combining AMF-friendly management practices, such as no-tillage with green manure mulching (NTG), is critical for mitigating nitrogen leaching and gaseous losses. Lyu et al. [42] found that NTG significantly increases AMF abundance and root colonization, which in turn improves soil-aggregate-associated nitrogen retention, optimizes root architecture for superior N uptake, increases *nosZ* gene abundance and N_2_O reductase activity, and effectively shifts the microbial balance to favor the conversion of N_2_O into harmless N_2_.

Mycorrhizal fungi are also expected to continue mitigating greenhouse gas emissions under future warming scenarios, as the experiments conducted on pasture species (*Medicago sativa* and *Festuca arundinacea*) at elevated temperatures (+4 °C) demonstrated that *Rhizophagus irregularis*’s ability to suppress nitrous oxide (N_2_O) fluxes is unaffected by elevated temperatures [43]. This highlights the importance of mycorrhizal fungi as a possible biological tool for mitigating the long-term effects of climate change.

However, the efficacy of AMF in mitigating N_2_O emissions sometimes yields neutral or inconsistent results. For instance, Okiobe et al. [44] conducted a mesocosm study that revealed that the potential N_2_O emissions did not change with *Rhizophagus irregularis* inoculation, as N_2_O potential emissions were driven predominantly by plant diversity rather than the AM-fungus. The increased AMF densities in the soil reduced the denitrification ratio and N_2_O production by denitrifiers, while the plant diversity reduced N_2_O potential emissions. This discrepancy highlights the need for shifting from controlled mesocosm experiments to large-scale open field experiments, which is essential to understand the function of AMF under fluctuating environmental variables, plant-microbe interactions, and agricultural management practices.

## 8. N_2_O Emission into Salinity-Affected Soils

Soil salinity has a direct impact on nitrous oxide (N_2_O) emissions and the efficiency of nitrogen utilization in soil [45]. Currently, salinity is a big problem for farmers in many regions of the world, accounting for around 7% of the Earth’s land surface, or over 1.1 billion hectares [46]. Climate change leads to increased soil salinity due to its effects on rising temperatures, increased evaporation, decreased rainfall, and acid rain [47]. Regardless of climate change, poor soil management caused by anthropogenic activities such as the excessive use of chemical fertilizers can contribute to the accumulation of soil salts over time. Salinity causes damage to both plants and soil, affecting plant physiological indices such as osmotic stress, nutritional imbalance, and poor water absorption, thus decreasing plant growth and productivity [48]. Salinity also affects soil microorganisms in terms of their abundance, diversity, and functions [49,50], and thus poor soil sustainability in the long term. Ultimately, salinity alters plant, soil, and microbial systems, which in turn modifies the N-cycle depending on the level of salinity.

Soil salinity and nitrous oxide (N_2_O) emissions have a complex and nonlinear relationship that is mostly determined by soil salinity levels. Nitrous oxide emissions typically increase at low to moderate salinity levels, as salinity at these levels inhibits nitrite oxidation over ammonia oxidation, increasing nitrous oxide production. This response is driven by specific biochemical shifts [7]: (1) increasing nitrate-reducing microbial communities, (2) inhibition of N_2_O reductase activity, and (3) increasing C and N availability by suppressing microbial respiration and then producing more N_2_O by nitrification and denitrification processes. On the other side, high salinity levels have a major toxic effect on the soil nitrogen mineralization, ammonification, nitrification, and indigenous microbial community, significantly impacting soil metabolism and may result in a decrease in gas flow [51].

For instance, in coastal wetland ecosystems, a salinity-affected soil [51] found that nitrogen enrichment drives a massive linear increase in N_2_O emissions, increasing by up to 848% through the denitrification pathway. However, the addition of exogenous salts shifts this response to a non-linear pattern, which confirms its inhibitory effect on microbial activity. This impact is further elucidated by the study of Li et al. [45], who found that N_2_O emissions reached their peak at electrical conductivity between 1.01 and 2.02 dS m^−1^, in which case the inhibition of nitrite oxidation occurs to a greater extent than that of ammonia oxidation. Similarly, Wei et al. [52] found that irrigation with brackish water at a concentration of 5 g L^−1^ increased N_2_O emissions by 87.7% compared to the control, while lower (2 g L^−1^) or higher (8 g L^−1^) concentrations reduced the emissions by 22.7% and 39.6%, respectively, compared to the control.

Taken together, these investigations show that moderate salinization causes a metabolic imbalance in the soil nitrogen cycle, which increases N_2_O emissions. Therefore, practical solutions for reducing N_2_O emissions in saline soils have been explored, including the use of microbial inoculants, which could be a useful strategy for mitigating these emissions. While the mechanism of action of these inoculations is not yet fully understood, the results are very promising. A recent study by Li et al. [14] found that using *Bacillus subtilis* biofertilizer in saline-alkali soil reduced N_2_O emissions by roughly 39%. This mitigation was linked to the suppression of nitrification-related N_2_O production pathway while maintaining crop productivity (Huanong 658 maize).

However, the link between microbial inoculants and N_2_O emissions in saline-affected soils is still fully unknown. Although the previous results of Li et al. [14] are very promising; however, due to the scarcity of field experiments, drawing broader conclusions about salinity-affected soils is difficult. Furthermore, the link between nitrogen addition and N_2_O emissions under salinity remains unclear. Hence, more research is needed to test more microbial strains and to understand how nitrogen inputs impact N_2_O emissions in salt-affected soils.

## 9. Conclusions

Reducing N_2_O emissions from agricultural soil is a significant problem for increasing nitrogen use efficiency while also protecting the environment in the face of climate change. This review discusses an environmentally benign biological solution: using microbial inoculants, such as bacteria and mycorrhizal fungi, to reduce N_2_O emissions from agricultural soils. Bacterial inoculants can help in the mitigation of N_2_O through both direct and indirect strategies. Direct mitigation relies on inoculation with N_2_O-reducing bacteria that contain the *nos*Z gene, which encodes the nitrous oxide reductase (N_2_OR) enzyme to convert N_2_O to harmless N_2_ while outcompeting native denitrifiers. The indirect strategy includes inoculation with non-N_2_O-reducing bacteria to alter the structure and activity of the native microbial community. This shift mitigates N_2_O production by reducing the (*amoA* + *amoB*)/(*nosZI* + *nosZII*) and (*nirS* + *nirK*)/(*nosZI* + *nosZII*) gene ratios. Similarly, arbuscular mycorrhizal fungi (AMF) inoculants mitigate N_2_O emissions from soils by improving nitrogen use efficiency through the enhanced root architecture and hyphae extension while effectively sequestering ammonia (NH_4_^+^). This deprives native nitrifiers of substrate, shifting the soil denitrification towards a lower (*nirS* + *nirK*)/*nosZ*) gene ratio, which promotes N_2_O consumption. Furthermore, these microbial inoculants could be used to mitigate N_2_O emissions in salt-affected soils. This is particularly critical because low to moderate salinity significantly increases N_2_O emissions, as the salts at these levels inhibit nitrite oxidation over ammonia oxidation. A promising field experiment shows a decrease in N_2_O emissions by suppressing the nitrification-related N_2_O production pathway in a maize field after *Bacillus subtilis* inoculation.

Future research should aim for a deeper understanding of the site’s features, including soil type, physical properties, and microbial diversity, to design tailored microbial inoculants that are effective against the specific soil characteristics under inquiry. Crucially, most research relies mainly on controlled greenhouse or microcosm environments; long-term field experiments are essential to validate these findings under variable soil conditions and climates. Further research is essential to fully understand the whole mechanism of mitigating N_2_O emission driven by the microbial inoculants in salinity-affected soil, which is not yet completely understood. Additionally, understanding how nitrogen input interacts with salinity to influence N_2_O dynamics represents a critical knowledge gap that requires further study. In this approach, addressing these knowledge gaps will allow the microbial inoculants to serve as a foundation for attaining sustainable agriculture, maintaining food security, and conserving the environment in the face of climate change.

## Figures and Tables

**Table 1:** Bacterial-inoculant-mediated N_2_O mitigation strategies and their underlying mechanisms.

Bacterial Strain	Crop/Soil Type	Mitigation Strategy	N_2_O Reduction	Mechanism of Mitigation	Reference
*Bradyrhizobium japonicum* (*nosZ*+/*nosZ*++)	Soybean	Direct	High (Field scale)	Reduction of N_2_O from decomposing root nodules via enhanced N_2_O reductase (N_2_OR)	[34]
*Bradyrhizobium diazoefficiens* (USDA 110 group)	Soybean	Direct	High (Field scale)	Utilization of indigenous mixed culture of *B*. *diazoefficiens* (*nosZ*+ strains) to consume postharvest N_2_O	[35]
*Bradyrhizobium* sp. G49	Soybean	Direct	70%	Selection of *nosZ*+ strains over *nosZ*− for efficient N_2_O to N_2_ conversion	[36]
*Dyadobacter fermentans*	Diverse Soils (11 types)	Direct	Up to 189%	Non-denitrifying bacteria acting as N_2_O sink via *nosZ*II-type reductase.	[37]
*Stutzerimonas stutzeri* NRCB010 and NRCB025	Tomato/Coarse and Fine Soil	Indirect	38.7–76.6%	Decreasing (*amoA* + *amoB*)/(*nosZI* + *nosZII*) and (*nirS + nirK*)/(*nosZ*I + *nosZ*II) ratios to favor complete denitrification	[38]
*Paenibacillus polymyxa*	Tea/Orchard Soil	Indirect	36.5–73.1%	Stimulation of *nirK* and *nosZ* genes, accelerating the conversion of N_2_O into harmless N_2_ gas	[39]
*Bacillus amyloliquefaciens*	Vegetables/Acidic Soil	Indirect	50.0%	Dual Action: Inhibiting nitrification by reduction of ammonia-oxidizing bacteria (AOB) and enhancing denitrification by increasing the abundance of N_2_O-reducing bacteria	[40]

**Table 2:** AMF-inoculant-mediated N_2_O mitigation strategies and their underlying mechanisms.

AMF Species/Strains	Agricultural Management/Climate Variable	Key Findings and Mechanisms	Impact on N_2_O/N-Cycling	Reference
*Rhizophagus intraradices* BJ09 and *Funneliformis mosseae* BJ04A	Biochar amendment in maize systems	Synergy between AMF and biochar increased biomass (19.23%) and reduced soil inorganic N (75.56%).	Suppressed N_2_O by reducing (*nirS* + *nirK*)/*nosZ*) gene ratio.	[31]
Indigenous AMF communities	Large-scale No-tillage and Green Manure (NTG)	NTG increased AMF colonization, improved soil aggregate N retention, and optimized root architecture.	Enhanced N_2_O reductase activity and reduced N leaching/gaseous losses.	[42]
*Rhizophagus intraradices*	^15^N isotope labeling in maize	AMF optimized root morphology to enhance N uptake, particularly under ammonium-based fertilization.	Reduced N_2_O through synergistic pathways: root expansion to enhance N uptake and altering the community of soil denitrifiers to favor a lower (*nirK* + *nirS*)/*nosZ* gene ratio.	[41]
Native AMF (Semiarid grassland)	Temperate Grasslands (Precipitation gradient)	AMF acted as a biological buffer across various rainfall levels (except extreme drought).	Lowered available N and shifted bacterial community towards N_2_O consumption (high *nosZ*).	[11]
*Rhizophagus irregularis*	Pasture Species and Warming (+4 °C)	AMF (*R. irregularis*) suppressed N_2_O emissions under both ambient and elevated temperatures.	Confirmed that AMF ecosystem services persist under future climate warming scenarios.	[43]
Mixed AMF communities (Indigenous)	AMF Hyphae and Organic patches	AMF hyphae effectively sequester ammonium (NH_4_^+^) from soil patches.	Reduced nitrification-driven N_2_O by outcompeting nitrifiers for substrate.	[9]
*Rhizophagus irregularis*	Plant diversity gradient under high soil fertility settings (Mesocosm)	AMF inoculation combined with diverse plant species split roles in N-cycling: Plant diversity reduced N_2_O emissions while AMF reduced the denitrification ratio	Reduced the denitrification ratio and N_2_O production by denitrifiers but had a neutral effect on actual potential N_2_O emissions	[44]

## Data Availability

No new data were created or analyzed in this study. Data sharing is not applicable to this article.

## References

[1] Ravishankara A.R., Daniel J.S., Portmann R.W. (2009). Nitrous Oxide (N_2_O): The Dominant Ozone-Depleting Substance Emitted in the 21st Century. Science.

[2] Montzka S.A., Dlugokencky E.J., Butler J.H. (2011). Non-CO_2_ greenhouse gases and climate change. Nature.

[3] Lan X., Thoning K.W., Dlugokencky E.J. (2026). Trends in Globally-Averaged CH_4_, N_2_O, and SF6 Determined from NOAA Global Monitoring Laboratory Measurements. Version 2026-06. https://gml.noaa.gov/ccgg/trends_doi.html.

[4] Syakila A., Kroeze C. (2011). The global nitrous oxide budget revisited. Greenh. Gas. Meas. Manag..

[5] Hu H.-W., Chen D., He J.-Z. (2015). Microbial regulation of terrestrial nitrous oxide formation: Understanding the biological pathways for prediction of emission rates. FEMS Microbiol. Rev..

[6] Zhang L., Song L., Wang B., Shao H., Zhang L., Qin X. (2018). Co-effects of salinity and moisture on CO_2_ and N_2_O emissions of laboratory-incubated salt-affected soils from different vegetation types. Geoderma.

[7] Yu Y., Zhao C., Zheng N., Jia H., Yao H. (2019). Interactive effects of soil texture and salinity on nitrous oxide emissions following crop residue amendment. Geoderma.

[8] Xiong R., Gao N., Huang W., Zhang X., Shen W. (2025). Recent progress in microbial production and consumption of nitrous oxide in agricultural soils. World J. Microbiol. Biotechnol..

[9] Storer K., Coggan A., Ineson P., Hodge A. (2018). Arbuscular mycorrhizal fungi reduce nitrous oxide emissions from N_2_O hotspots. New Phytol..

[10] Govindarajulu M., Pfeffer P.E., Jin H., Abubaker J., Douds D.D., Allen J.W., Bücking H., Lammers P.J., Shachar-Hill Y. (2005). Nitrogen transfer in the arbuscular mycorrhizal symbiosis. Nature.

[11] Li J., Meng B., Yang X., Cui N., Zhao T., Chai H., Zhang T., Sun W. (2022). Suppression of AMF accelerates N_2_O emission by altering soil bacterial community and genes abundance under varied precipitation conditions in a semiarid grassland. Front. Microbiol..

[12] Li Y., Xu J., Liu S., Qi Z., Wang H., Wei Q., Gu Z., Liu X., Hameed F. (2020). Salinity-induced concomitant increases in soil ammonia volatilization and nitrous oxide emission. Geoderma.

[13] Reddy N., Crohn D.M. (2014). Effects of soil salinity and carbon availability from organic amendments on nitrous oxide emissions. Geoderma.

[14] Li R., Lin X., Miao Y., Zhang C., Li F., Zhang G., Sun Q., Hua T., Wang J. (2026). *Bacillus subtilis* Biofertilizer Mitigates N_2_O Emissions from Saline-Alkali Farmland. Life.

[15] Van Kessel M.A.H.J., Speth D.R., Albertsen M., Nielsen P.H., Op den Camp H.J.M., Kartal B., Jetten M.S.M., Lücker S. (2015). Complete nitrification by a single microorganism. Nature.

[16] Daims H., Lebedeva E.V., Pjevac P., Han P., Herbold C., Albertsen M., Jehmlich N., Palatinszky M., Vierheilig J., Bulaev A. (2015). Complete nitrification by Nitrospira bacteria. Nature.

[17] Jin D., Zhang X., Zhang X., Zhou L., Zhu Z., Deogratias U.K., Wu Z., Zhang K., Ji X., Ju T. (2024). A critical review of comammox and synergistic nitrogen removal coupling anammox: Mechanisms and regulatory strategies. Sci. Total Environ..

[18] Zumft W.G. (1997). Cell biology and molecular basis of denitrification. Microbiol. Mol. Biol. Rev..

[19] Wallenstein M.D., Myrold D.D., Firestone M., Voytek M. (2006). Environmental controls on denitrifying communities and denitrification rates: Insights from molecular methods. Ecol. Appl..

[20] Kuenen J.G. (2008). Anammox bacteria: From discovery to application. Nat. Rev. Microbiol..

[21] Conthe M., Wittorf L., Kuenen J.G., Kleerebezem R., van Loosdrecht M.C.M., Hallin S. (2018). Life on N_2_O: Deciphering the ecophysiology of N2O respiring bacterial communities in a continuous culture. ISME J..

[22] Lin Y., Hu H.-W., Deng M., Yang P., Ye G. (2023). Microorganisms carrying nosZ I and nosZ II share similar ecological niches in a subtropical coastal wetland. Sci. Total Environ..

[23] Li H., Luo Q., Sun L., Xu H., Hao X., Zhu K., Li M., Li B., Jiao W., Geng J. (2025). Comparative study of nosZI and nosZII clade isolates: Insights into their responses to environmental variables and soil fertilization types. Front. Plant Sci..

[24] Edenhofer O. (2015). Climate Change 2014: Mitigation of Climate Change.

[25] Luo Z., Lam S.K., Fu H., Hu S., Chen D. (2019). Temporal and spatial evolution of nitrous oxide emissions in China: Assessment, strategy and recommendation. J. Clean. Prod..

[26] Kuypers M.M.M., Marchant H.K., Kartal B. (2018). The microbial nitrogen-cycling network. Nat. Rev. Microbiol..

[27] Zhu G., Shi H., Zhong L., He G., Wang B., Shan J., Han P., Liu T., Wang S., Liu C. (2025). Nitrous oxide sources, mechanisms and mitigation. Nat. Rev. Earth Environ..

[28] Conthe M., Lycus P., Arntzen M.Ø., Ramos da Silva A., Frostegård Å., Bakken L.R., Kleerebezem R., van Loosdrecht M.C.M. (2019). Denitrification as an N_2_O sink. Water Res..

[29] Han B., Yao Y., Liu B., Wang Y., Su X., Ma L., Liu D., Niu S., Chen X., Li Z. (2024). Relative importance between nitrification and denitrification to N2O from a global perspective. Glob. Change Biol..

[30] Guo J., Liang X., Lei W., Zhang Z., Shen Y., Han S., Wang H., Qian Y., Nie B., Wang L. (2026). Contrasting microbial sources of soil N2O emissions revealed by metagenomics in natural and agricultural soils along the Yellow River. Environ. Res..

[31] Wang Y., Liu J., Deng X., Li Y., Gao J., Liu L. (2024). Arbuscular Mycorrhizae Affect Soil Nitrogen Fertilizer Utilization, Denitrification Functional Genes, and N_2_O Emissions During Biochar Amendment. Agronomy.

[32] Jones C.M., Spor A., Brennan F.P., Breuil M.-C., Bru D., Lemanceau P., Griffiths B., Hallin S., Philippot L. (2014). Recently identified microbial guild mediates soil N2O sink capacity. Nat. Clim. Change.

[33] Dăncilă A.M., Modrogan C., Orbuleț O.D. (2025). Effects of Nitrogen Fertilizer Application on N_2_O Emissions from Rice Cultivation: A Review. Environments.

[34] Itakura M., Uchida Y., Akiyama H., Hoshino Y.T., Shimomura Y., Morimoto S., Tago K., Wang Y., Hayakawa C., Uetake Y. (2013). Mitigation of nitrous oxide emissions from soils by Bradyrhizobium japonicum inoculation. Nat. Clim. Change.

[35] Akiyama H., Hoshino Y.T., Itakura M., Shimomura Y., Wang Y., Yamamoto A., Tago K., Nakajima Y., Minamisawa K., Hayatsu M. (2016). Mitigation of soil N_2_O emission by inoculation with a mixed culture of indigenous *Bradyrhizobium diazoefficiens*. Sci. Rep..

[36] Hénault C., Barbier E., Hartmann A., Revellin C. (2022). New Insights into the Use of Rhizobia to Mitigate Soil N_2_O Emissions. Agriculture.

[37] Domeignoz-Horta L.A., Putz M., Spor A., Bru D., Breuil M.C., Hallin S., Philippot L. (2016). Non-denitrifying nitrous oxide-reducing bacteria—An effective N_2_O sink in soil. Soil Biol. Biochem..

[38] Gao N., Zhang H., Hu C., Li Q., Li L., Lei P., Xu H., Shen W. (2024). Inoculation with Stutzerimonas stutzeri strains decreases N_2_O emissions from vegetable soil by altering microbial community composition and diversity. Microbiol. Spectr..

[39] Zhou S., Zeng X., Xu Z., Bai Z., Xu S., Jiang C., Zhuang G., Xu S. (2019). *Paenibacillus polymyxa* biofertilizer application in a tea plantation reduces soil N_2_O by changing denitrifier communities. Can. J. Microbiol..

[40] Wu S., Zhuang G., Bai Z., Cen Y., Xu S., Sun H., Han X., Zhuang X. (2018). Mitigation of nitrous oxide emissions from acidic soils by Bacillus amyloliquefaciens, a plant growth-promoting bacterium. Glob. Change Biol..

[41] He T., Lin W., Yang S., Du J., Giri B., Feng C., Gilliam F.S., Zhang F., Zhang X., Zhang X. (2024). Arbuscular mycorrhizal fungi reduce soil N2O emissions by altering root traits and soil denitrifier community composition. Sci. Total Environ..

[42] Lyu H., Yu A., Chai Q., Wang Y., Wang F., Wang P., Shang Y. (2024). Arbuscular mycorrhizal fungi mediate soil N dynamics, mitigating N_2_O emissions and N-leaching while promoting crop N uptake in green manure systems. Sci. Total Environ..

[43] Zhang H., Powell J.R., Power S.A., Churchill A.C., Plett J.M., Macdonald C.A., Jacob V., Kim G.W., Pendall E., Tissue D.T. (2021). Arbuscular mycorrhizal fungal-mediated reductions in N_2_O emissions were not impacted by experimental warming for two common pasture species. Pedobiologia.

[44] Okiobe S.T., Rillig M.C., Mola M., Augustin J., Parolly G., Veresoglou S.D. (2020). Arbuscular mycorrhiza has little influence on N2O potential emissions compared to plant diversity in experimental plant communities. FEMS Microbiol. Ecol..

[45] Li Y., Xu J., Liu B., Wang H., Qi Z., Wei Q., Liao L., Liu S. (2020). Enhanced N2O Production Induced by Soil Salinity at a Specific Range. Int. J. Environ. Res. Public Health.

[46] FAO (2021). Global Map of Salt-Affected Soils.

[47] Muhammad M., Waheed A., Wahab A., Majeed M., Nazim M., Liu Y.-H., Li L., Li W.-J. (2024). Soil salinity and drought tolerance: An evaluation of plant growth, productivity, microbial diversity, and amelioration strategies. Plant Stress.

[48] Shrivastava P., Kumar R. (2015). Soil salinity: A serious environmental issue and plant growth promoting bacteria as one of the tools for its alleviation. Saudi J. Biol. Sci..

[49] Zaki R.M., Afify A.H., Ashour E.H., El-Sawah A.M. (2025). Salt-Tolerant Bacteria Support Salinity Stress Mitigating Impact of Arbuscular Mycorrhizal Fungi in Maize (*Zea mays* L.). Microorganisms.

[50] El-Sawah A.M., Zaki R.M., Ashour E.H., Afify A.H. (2026). The synergistic effect of salt-tolerant *Stutzerimonas stutzeri* and arbuscular mycorrhizal fungi alleviates salinity stress in soybean (*Glycine max*, L.). World J. Microbiol. Biotechnol..

[51] Jia J., Bai J., Wang W., Yin S., Zhang G., Zhao Q., Wang X., Liu X., Cui B. (2020). Salt stress alters the short-term responses of nitrous oxide emissions to the nitrogen addition in salt-affected coastal soils. Sci. Total Environ..

[52] Wei Q., Li X., Xu J., Dai H., Li B., Xu J., Wei Q., Wang K. (2022). Responses of Soil N_2_O and CO_2_ Emissions and Their Global Warming Potentials to Irrigation Water Salinity. Atmosphere.

